# Label-free multiphoton imaging allows brain tumor recognition based on texture analysis—a study of 382 tumor patients

**DOI:** 10.1093/noajnl/vdaa035

**Published:** 2020-03-12

**Authors:** Ortrud Uckermann, Roberta Galli, Georg Mark, Matthias Meinhardt, Edmund Koch, Gabriele Schackert, Gerald Steiner, Matthias Kirsch

**Affiliations:** 1 Neurosurgery, University Hospital Carl Gustav Carus, TU Dresden, Dresden, Germany; 2 Clinical Sensoring and Monitoring, Department of Anesthesiology and Intensive Care Medicine, Faculty of Medicine, TU Dresden, Dresden, Germany; 3 Neuropathology, Institute of Pathology, University Hospital Carl Gustav Carus, TU Dresden, Dresden, Germany

**Keywords:** brain metastases, intraoperative, label-free imaging, primary brain tumors, tumor delineation

## Abstract

**Background:**

Label-free multiphoton microscopy has been suggested for intraoperative recognition and delineation of brain tumors. For any future clinical application, appropriate approaches for image acquisition and analysis have to be developed. Moreover, an evaluation of the reliability of the approach, taking into account inter- and intrapatient variability, is needed.

**Methods:**

Coherent anti-Stokes Raman scattering (CARS), two-photon excited fluorescence (TPEF), and second-harmonic generation were acquired on cryosections of brain tumors of 382 patients and 28 human nontumor brain samples. Texture parameters of those images were calculated and used as input for linear discriminant analysis.

**Results:**

The combined analysis of texture parameters of the CARS and TPEF signal proved to be most suited for the discrimination of nontumor brain versus brain tumors (low- and high-grade astrocytoma, oligodendroglioma, glioblastoma, recurrent glioblastoma, brain metastases of lung, colon, renal, and breast cancer and of malignant melanoma) leading to a correct rate of 96% (sensitivity: 96%, specificity: 100%). To approximate the clinical setting, the results were validated on 42 fresh, unfixed tumor biopsies. 82% of the tumors and, most important, all of the nontumor samples were correctly recognized. An image resolution of 1 µm was sufficient to distinguish brain tumors and nontumor brain. Moreover, the vast majority of single fields of view of each patient’s sample were correctly classified with high probabilities, which is important for clinical translation.

**Conclusion:**

Label-free multiphoton imaging might allow fast and accurate intraoperative delineation of primary and secondary brain tumors in combination with endoscopic systems.

**Key Points**

- Automated analysis of label-free multiphoton images discerns neoplastic and nontumor brain.
- Primary and secondary brain tumors are recognized with high accuracy.
- Clinical translation using endoscopic systems will allow intraoperative tumor delineation.

**Importance of the Study**

Research of the recent years suggests a great potential of label-free multiphoton imaging for intraoperative recognition of brain tumors. However, for a future clinical translation, appropriate strategies for obtaining a reliable diagnostic readout have to be developed. Here, we show that automated image analysis, specifically texture analysis, extracts key features that allow classification of brain tumors versus nontumor brain. The approach enabled to discern brain tumors ranging from low- to high-grade glioma and recurrent glioblastoma to brain metastases without prior knowledge of tumor type. This suggests that the changes in morphochemistry that are induced by malignant transformation are reflected by general alterations of image features. High probabilities for tissue classification were confirmed and analysis of multiple images of the samples gave consistent results underlining the stability of the approach and its reliability for future clinical exploitation using endoscopic systems.

The standard treatment for brain tumors is surgical resection followed by adjuvant therapies. The extent of resection is directly related to the patient’s progression-free and overall survival for glioma.^1–3^ For patients with brain metastases of peripheral cancers the situation is more complex, as survival is largely affected by the primary tumor disease. However, gross total resection has beneficial effects.^4,5^ Therefore, neurosurgery needs precise tools for intraoperative tumor recognition to offer safe brain tumor surgery with the optimized extent of resection.

Brain tumor resection is carried out essentially based on optical information provided by the surgical microscope, the tactile sense, and the knowledge of anatomy of the neurosurgeon. Planning of surgical approaches and localization of tumors is obtained using neuronavigation, which, however, is of limited use for tumor delineation because of the intraoperative brain shift. Further improvement of the extent of resection is achieved by intraoperative imaging using fluorescent markers like 5-aminolevulinic acid (5ALA) or fluorescein, intraoperative MRI, or ultrasound.^6,7^ Stimulation mapping and monitoring techniques are used to identify and preserve eloquent areas and the functional integrity of neural pathways. However, the tools available for intraoperative imaging have some limitations: Intraoperative MRI enables to increase the extent of resection, but this technology is restricted to large centers because of immense costs and constructional requirements. 5ALA is only approved for high-grade glioma. It is not recommended for low-grade glioma as the majority of these lesions lack substantial 5ALA fluorescence. Approximately 50% of brain metastases can be visualized by 5ALA fluorescence.^8^ Fluorescein can be employed for brain tumor visualization in case of impaired blood–brain barrier integrity. However, it is not a selective tumor marker and not approved for fluorescence-guided tumor resection. For any fluorescence-based technology, the kinetics of administration, accumulation, metabolism, and degradation have to be taken into account and fluorophores can be destroyed or bleached.^6,9^

During the last years, innovative approaches using label-free multiphoton imaging technologies were investigated for intraoperative tissue analysis. Coherent anti-Stokes Raman scattering (CARS) microscopy is a technology that is usually tuned to address the Raman band at 2850 cm^-1^ generated by C–H bond vibrations in the tissue. Therefore, it mainly visualizes the distribution of lipids in the brain, showing for example myelin sheaths as well as lipid droplets with high contrast. Moreover, it reveals the overall tissue structure based on the signal of CH_x_ groups of proteins. Analyses of the CARS signal showed lower intensities in both primary and secondary brain tumors than in gray (and of course in white) matter.^10,11^ This was confirmed in vivo on orthotropic glioma in the mouse model.^12^ Besides CARS, two-photon excited fluorescence (TPEF) of endogenous fluorophores and second-harmonic generation (SHG) generated mainly by fibrillary collagen can be simultaneously acquired. The combination of different label-free modalities substantially enriches the morphochemical information of label-free microscopic images and proved to show cytological and architectural features in single fields of view that are the basis for histopathology.^13–15^

All previous studies support the hypothesis that brain tumors and normal brain tissue can be discerned based on label-free multiphoton imaging. Experimental and human glioma were shown to have a specific morphochemistry in the CARS images, with enlarged nuclei bearing a pronounced nuclear membrane and visible nucleolus.^12^ Moreover, several features in label-free multiphoton images that are limited to brain pathologies have been identified^15^ and might be employed as tumor markers. Preliminary data on 55 lesions of the central nervous system indicated that information required for pathological tumor typing and grading can principally be retrieved by label-free multiphoton microscopy.^13^ Moreover, automated segmentation provided objective data on cell density and nuclei in human brain tumor samples, both information being diagnostically relevant.^16^

In the present study, we aim to provide the basis for clinical translation of label-free multiphoton imaging as an intraoperative tool for brain tumor delineation. Therefore, we addressed a large cohort of different types of human brain tumors including the analysis of fresh biopsies and focused on the analysis of single fields of view. Texture analysis, which quantifies image texture as functions of the spatial variation in pixel intensities, has been successfully employed for image analysis of histological and CARS datasets of various types of cancer.^17,18^ Therefore, this approach was employed in combination with classification to extract clinically relevant information from label-free multiphoton images of brain tumors in an observer-independent manner.

## Materials and Methods

### Tissue Samples

Biopsies of human brain tumors and nontumor samples of sclerotic hippocampi were obtained during routine surgery. The study included samples of low-grade astrocytoma WHO I/II (*n* = 14), anaplastic astrocytoma WHO III (*n* = 73), anaplastic oligodendroglioma WHO III (*n* = 41), glioblastoma (GBM) (*n* = 91), recurrent GBM (*n* = 18), brain metastases of lung cancer (*n* = 46), colon cancer (*n* = 25), renal cancer (*n* = 20), breast cancer (*n* = 24), prostate cancer (*n* = 1), and of malignant melanoma (*n* = 29). All patients gave written consent and the use of human tissue was approved by the ethics committee of the TU Dresden (EK 323122008). Additionally, samples of the nontumor brain were obtained from autopsy (anonymous body donation). In total, samples from 28 patients or body donations were available for the study. For the preparation of cryosections, the tissue was either snap-frozen in liquid nitrogen followed by embedding in cryomedium (Leica Biosystems Nussloch GmbH) or fixed in 4% formalin, dehydrated in 10% and 30% sucrose for 24 h, respectively, and then embedded in cryomedium. Cryosections of 10 µm were prepared and stored at −20°C until use.

For label-free multiphoton imaging, cryosections were rehydrated with phosphate-buffered saline (PBS). Fresh tissue was stored in PBS or isotonic NaCl and imaged without any processing within 30 min after removal.

### Label-Free Multiphoton Imaging

The multiphoton microscope has been described elsewhere.^19^ Two fiber lasers at 781 nm and at 1005 nm (FemtoFiber pro NIR and FemtoFiber pro TNIR both from Toptica Photonics AG) were used for excitation. CARS and SHG were acquired in transmission for sections and in reflection on fresh samples using emission filters 626–654 nm and 380–400 nm, respectively. TPEF was always acquired in reflection using a bandpass filter 500–550 nm. The imaging position was chosen on areas of solid tumor upon inspection of a hematoxylin and eosin stained consecutive section. The field of view (FoV) was set to 104 µm × 208 µm and was scanned with 104 × 208 pixels resulting in a pixel size of 1 µm and an averaging of 4. A larger area was scanned using a tiling procedure in order to analyze multiple FoVs of a sample. Usually, 100 FoVs were obtained from each sample; the minimum number acquired was 24 FoVs on a sample containing a small region of the nontumor brain at the tumor border.

### Texture Analysis and Classification

Analysis of each FoV was performed using MATLAB software (The MathWorks Inc.). Each signal channel was analyzed separately. After min–max normalization, 13 texture parameters were calculated using MATLAB functions. First-order parameters included mean gray value, standard deviation, kurtosis, skewness, and entropy. Furthermore, second-order parameters were determined. Gray-level co-occurrence matrices were calculated in 4 orientations (0°, 45°, 90°, and 135°) and used to obtain values for contrast, correlation, energy, and homogeneity for 2 different distances (6 µm and 40 µm). For each distance, texture parameters were calculated as averages of the 4 orientations.

For analysis of brain tumors versus nontumor brain on cryosections and fresh tissue, the dataset was split by random assignment of patients to test and training set. All FoVs of each patient (usually 100) were used to build the training set (177 brain tumors, 14 nontumor brain tissue) and the independent test set (204 brain tumors, 14 nontumor brain samples, Supplementary Table S1). Texture parameters were then used for linear discriminant analysis (MATLAB function “classify”). The classification function provided a probability of class assignment (0 = nontumor, 1 = tumor) for each channel of every FoV. For combined CARS + TPEF analysis, the probability of class assignment was calculated based on the analysis of texture parameters of both channels (ie, of 26 texture parameters).

Finally, the median value of the probabilities of class assignment of all FoVs was calculated for each patient (cryosections) or sample (fresh tissue) and plotted using a color code ranging from blue (nontumor) to red (tumor) to obtain a diagnostic rating.

Image analysis was conducted using MATLAB R2018b on a standard PC (Intel Core i7-6700 CPU 3.4 GHz). Processing of a FoV (100 × 208 pixels, 2 channels: TPEF and CARS) including normalization and calculation of texture parameters required approximately 50 ms. It resulted in 26 values that are then used as input for classification, which required approximately 60 ms using the training set for cryosections.

Histograms were plotted using GraphPad Prism 6.0 (GraphPad Software Inc.).

## Results

In this study, we acquired images of brain tumors or non-neoplastic brain using label-free multiphoton microscopy. Starting on tissue cryosections, primary human brain tumors and brain metastases of peripheral cancers were investigated. Texture analysis was employed to transform the complex information contained in each image into a simple set of values. Thirteen texture parameters were calculated for each channel (CARS, TPEF, and SHG) and then used to classify tissue types based on linear discriminant analysis.

**Figure 1.**
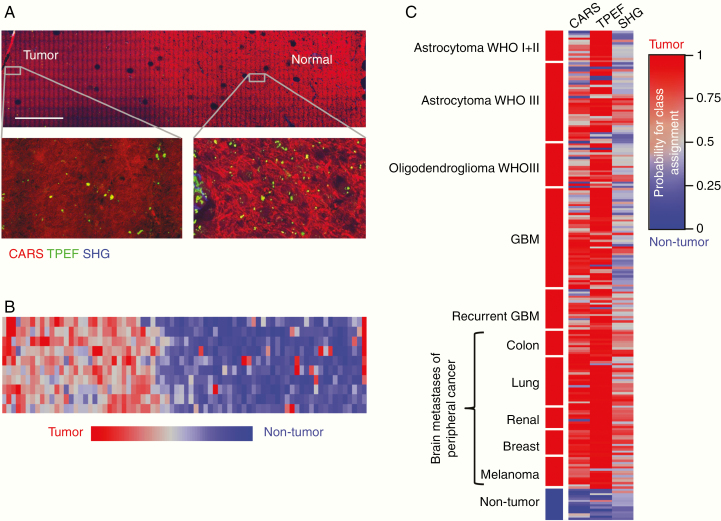
Classification of brain tumors versus nontumor brain tissue based on CARS, TPEF, or SHG. (A) CARS/TPEF/SHG image of the tumor border of an anaplastic astrocytoma WHO III, scale bar 1 mm. (B) False-color image of the probability of class assignment based on texture analysis of CARS images for the sample shown in A. (C) Probability of class assignment plotted for each patient of the test set using a color code ranging from red (tumor) to blue (nontumor) for the CARS, TPEF, and SHG images, respectively. Each line represents 1 patient. Linear discriminant analysis of texture parameters of brain tumors (*n* = 204) versus nontumor brain tissue (*n* = 14) was performed using an independent training set.

Initially, we confirmed that “low-quality” images with a pixel dimension of 1 µm are suitable for the suggested approach (Supplementary Figure S1). Based on these findings, an FoV of 104 × 208 µm^2^ was always scanned with 104 × 208 points resulting in an acquisition time of 0.8 s and small file size enabling fast analysis. As the first potential clinical application, identification of tumor borders on label-free multiphoton imaging was addressed. A tissue section containing the border of an astrocytoma WHO III is shown in Figure 1A. A large tissue area was imaged by the acquisition of multiple FoVs using a tiling procedure. Here, differences between the microarchitecture of tumor and nontumor brain tissue can be appreciated (see magnifications in Figure 1A). Tissue classification based on texture parameters of CARS images was employed for visualization of the tumor border (Figure 1B). The probability of class assignment was calculated for each FoV and is displayed using a color code ranging from red (tumor) to blue (nontumor) using an independent training set (Supplementary Table S1). Nontumor brain areas are recognized with high probabilities and the transition toward the tumor is clearly visible in this example.

To further address clinical demands, we evaluated whether tissue classification based on texture parameters of label-free multiphoton images is suited for a more fundamental approach and tested the discrimination of the categories “brain tumor” versus “nontumor brain tissue.” Classification of texture parameters of all FoV images of each biopsy was performed and followed by a calculation of the probability of class assignment for each patient. Figure 1C shows the result of the classification of the test set using a color code, and the reclassification of the training set is shown in Supplementary Figure S2. The number of correctly classified patient’s biopsies is given in Table 1.

**Table 1.** Classification Result Based on Texture Parameters of CARS, TPEF, or SHG Images for Brain Tumors Versus Nontumor Brain Tissue

		Correctly Classified Biopsies		
Test Set	*n*	CARS	TPEF	SHG
Astrocytoma WHO I + II	14	9	14	3
Astrocytoma WHO III	36	28	28	19
Oligodendroglioma WHO III	20	15	19	7
GBM	45	39	44	15
Recurrent GBM	18	13	16	10
Metastasis of colon cancer	12	12	12	12
Metastasis of lung cancer	23	22	22	17
Metastasis of renal cancer	10	9	10	9
Metastasis of breast cancer	12	12	12	9
Metastasis of melanoma	14	13	13	10
Nontumor brain	14	13	12	13

The total number (*n*, 1 biopsy of each patient) and the number of correctly classified patient’s biopsies are given for the tumor types of the independent test set.

Analysis of texture parameters of CARS and TPEF images provided solid correct rates for classification of primary and secondary brain tumors, respectively. Based on CARS images, 172/204 brain tumors were assigned to the correct class while upon analysis of TPEF images 190/204 brain tumors were correctly classified. Information obtained by SHG allowed the detection of brain metastases of peripheral cancers (57/71) while it failed to recognize glioma (54/133).

We observed more often intermediate probabilities for a class assignment (around 0.5) for glioma than for metastases (gray colors in Figure 1C and Supplementary Figure S3). These patient’s biopsies can technically be assigned to either class, but the clinical relevance of such a result has to be critically considered.

Most samples of non-neoplastic tissue were assigned to the correct class based on texture parameters of either CARS, TPEF, or SHG images. While analysis of CARS or TPEF images resulted in high probabilities for a class assignment, intermediate probabilities were often obtained for analysis of SHG images (median CARS: 0.19, TPEF: 0.22, SHG: 0.39, Figure 1C and Supplementary Figure S3).

**Figure 2.**
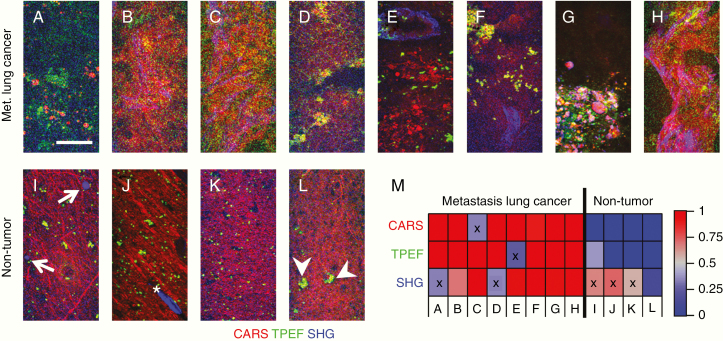
Micromorphology of human metastases of lung cancer and nontumor tissue. (A–H) CARS/TPEF/SHG images of metastases of lung cancer. (I–L) CARS/TPEF/SHG images of nontumor brain tissue. The intensity of channels was normalized (min–max), arrows indicate corpora amylacea, asterisk indicates a blood vessel, arrow heads indicate cells with fluorescent inclusions, scale bar: 50 µm. (M) Classification result of the images shown in A–I for each channel. The color code shows the probability of class assignment for being nontumor (blue) and metastasis of lung cancer (red); “x” indicates misclassification.

In order to relate texture analysis to tissue micromorphology, we inspected a subset of images and compared tissue features with the results of the classification. Figure 2A–H shows images of brain metastases of lung cancer of 8 patients and of non-neoplastic brain (Figure 2I–L) as an example. Note that there are no differences in the CARS signal intensities of gray and white matter as the intensities of images were normalized for calculation of texture parameters. The raw images are provided in Supplementary Figure S4 for reference.

The appearance of metastases visualized by CARS is highly inhomogeneous within a FoV, while nontumor tissue displays a more ordered structure of axons and cells. Texture analysis is sensitive to this pattern and classification resulted in the assignment of a metastasis image with a regular pattern of densely packed cells (Figure 2C) to the class “nontumor” (Figure 2M). In the TPEF channel, nontumor tissue is characterized by a rather regular distribution of small fluorescent structures. One image of lung metastases displays similar fluorescent structures and is in fact misclassified (Figure 2E). SHG-active structures were rare in nontumor brain tissue and limited to corpora amylacea (arrows in Figure 2I) and blood vessels (asterisk in Figure 2J), while SHG collagenous structures were often observed in metastases. As a consequence, texture analysis of the SHG channel leads to the false classification of nontumor tissue that contains SHG-active structures (Figure 2I and J) and of tumor lacking such structures (Figure 2A and D). Furthermore, the procedure of signal intensity normalization within the single FoV leads to the introduction of very high noise whenever SHG-active structures are lacking (Figure 2K), which in turn leads to intermediate classification probabilities.

Although the presented examples do not fully resume the relationship between tissue morphology and image texture, they clearly show how the 3 channels carry complementary information. This also enables a better understanding of the classification results in Figure 1C. Here, texture analysis of CARS is best in recognizing nontumor tissue and of TPEF in recognizing tumors. Texture analysis of SHG failed to classify tumors without extracellular matrix (ECM) alterations as well as nontumor tissue. Therefore, we tested whether merging CARS and TPEF texture parameter sets would improve the classification results.

**Figure 3.**
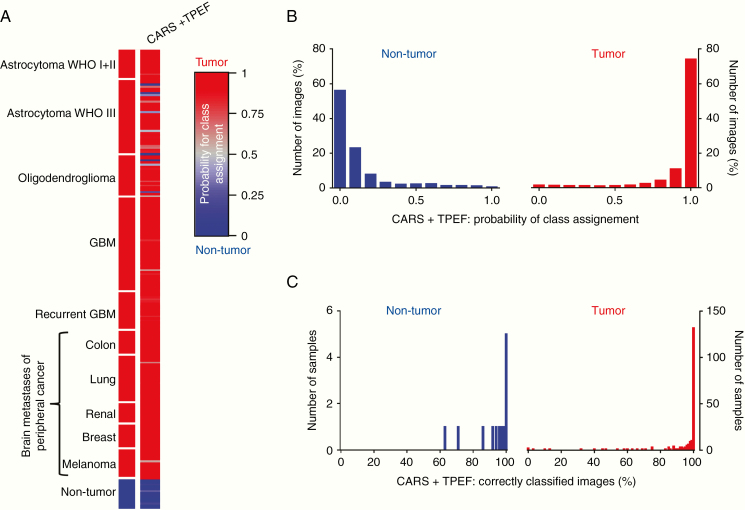
Classification result for brain tumors versus nontumor brain tissue based on combined analysis of CARS + TPEF. (A) Probability of class assignment is plotted for each patient of the test set for the combined analysis of texture parameters of CARS and TPEF images, respectively. (B) Distribution of the probability of class assignment for image classification based on combined CARS + TPEF analysis. (C) Percentage of correctly classified images for each sample based on combined CARS + TPEF analysis.

Indeed, the overall correct rate of combined analysis of CARS and TPEF images increased to 96% (Table 2) and all nontumor brain samples were correctly classified with improved probabilities of class assignment compared to single-channel analysis (median TPEF + CARS: 0.04, Supplementary Figure S3, orange symbols). Figure 3A indicates that only 6 astrocytic tumors and 3 oligodendrocytic tumors were misclassified of the 204 different tumors analyzed in the independent test set. All low-grade astrocytoma, all GBM, including recurrent GBM, and all brain metastases were correctly assigned with high probabilities for a class assignment. The reclassification of the training set is shown in Supplementary Figure S2. Analysis of the classification of the multiple FoVs that were obtained on each sample showed that the images were assigned to the correct group with high probabilities based on combined analysis of CARS + TPEF texture parameters (Figure 3B). Moreover, for the majority of samples (11/14 nontumor and 175/204 tumor samples), more than 90% of the FoVs acquired were assigned to the correct group, and actually all images were correctly classified for 5 nontumor and 132 tumor samples (Figure 3C).

**Table 2.** Sensitivity (Correct Rate Tumor), Specificity (Correct Rate Nontumor Brain), and Correct Rates for Classification of Brain Tumors Versus Nontumor Brain Tissue

Sensitivity			Specificity			Correct Rate		
CARS	TPEF	CARS + TPEF	CARS	TPEF	CARS + TPEF	CARS	TPEF	CARS + TPEF
84%	93%	96%	93%	86%	100%	85%	93%	96%

Results are based on texture parameters of CARS or TPEF images and combined analysis of CARS and TPEF (independent test set, tumors: *n* = 204 patient’s biopsies, nontumor brain tissue: *n* = 14).

**Figure 4.**
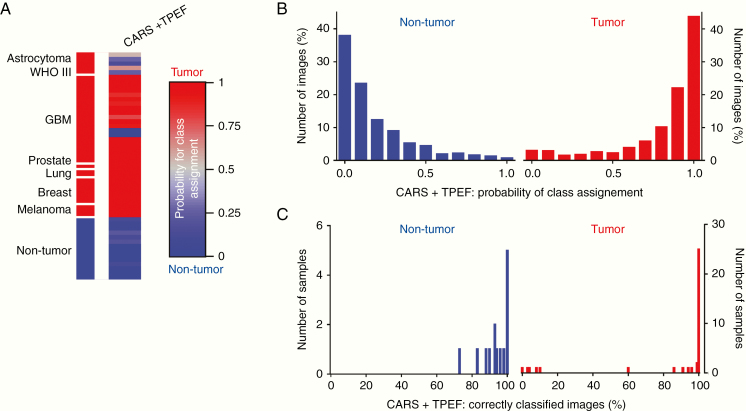
Classification result for fresh brain tumor biopsies versus nontumor brain tissue. (A) Probability of class assignment is plotted for each sample of the test set. Linear discriminant analysis of texture parameters of brain tumor biopsies (*n* = 37 samples of 11 patients) versus nontumor brain tissue (*n* = 14 samples of 10 patients/body donors) was performed using an independent training set. Results based on combined analysis of CARS + TPEF images. (B) Distribution of the probability of class assignment for image classification based on combined CARS + TPEF analysis. (C) Percentage of correctly classified images for each sample based on combined CARS + TPEF analysis.

Finally, we evaluated whether our approach can be translated to ex situ analysis of unprocessed, fresh tissue and analyzed 110 bulk samples of 42 patients/body donors. Figure 4A shows the classification result based on linear discriminant analysis of combined texture parameters of CARS and TPEF images of the independent test set. The reclassification of the training set is shown in Supplementary Figure S5. Note that some tumor entities were only represented in the training and not in the test set and vice versa because only samples of 1 patient were available.

Similar to the results obtained on cryosections, misclassified samples were found for astrocytoma WHO III. Five samples were obtained from 1 astrocytoma patient, among those, 3 samples were misclassified (sample 2, 3, 5, probability of class assignment: 0.27, 0.15, and 0.26, respectively) and 2 were correctly recognized as “tumor” although with intermediate probabilities (sample 1 and 4, probability of class assignment: 0.55 and 0.64, respectively). For all other patients, the analysis of different samples gave consistent results. However, we also found that all samples of 1 GBM patient were recognized as “nontumor” (2 samples: the probability of class assignment: 0.02 and 0.03, respectively). Here, misclassification might have been driven by the presence of fluorescent cells and the absence of pronounced tumor-induced alterations (compare example in Figure 2E). Taken together, 82% (9/11) of the tumors and, most important, all nontumor samples were correctly recognized, giving an overall correct rate of 90% for the classification of fresh, unfixed tissue samples based on combined analysis of CARS and TPEF texture parameters. The vast majority of images were classified with high probabilities (Figure 4B) and almost all images obtained on a sample were assigned to the correct group (Figure 4C) matching the results obtained on cryosections.

## Discussion

Label-free multiphoton imaging in synergy with texture analysis proved to be an excellent tool for an objective, observer-independent recognition of various types of brain tumors. The classification approach based on combined analysis of CARS and TPEF images resulted in overall 95% accuracy for discrimination of brain tumors and nontumor brain tissue. However, even the gold standard that is histopathology does not always deliver a definite diagnosis because of observer-related differences.^20^ Upon reviewing 500 cases of brain tumors, disagreement with the original diagnosis was found in 43%. Most important, major changes in diagnosis having significance for therapy or intervention were found in 9% of cases.^21^

All of the 3 modalities investigated can be potentially useful for discrimination of brain tumors and nontumor brain. However, analyses of CARS and TPEF images were overall more suited for tumor recognition than the analyses of SHG images, which is consistent with the differences in micromorphology.

In nontumor brain tissue, SHG originates from the collagen of the adventitia of blood vessels^22^ and from corpora amylacea.^23^ Only a few FoVs comprise those structures, while others do not have any SHG signal.^24^ Glioma might show substantial changes in this feature. Strong upregulation of the SHG signal due to aberrant vessels^25^ and due to a changed ECM with the deposition of collagen bundles^26^ has been reported. However, these changes in SHG display a high interpatient variability.^15,24^ Therefore, some gliomas are characterized by a pronounced SHG signal in most FoVs, while other tumor samples do not show any or just minor changes compared to nontumor brain. Brain metastases are known to preserve micromorphological features of the parental tumor and reorganization of collagen being a major component of the ECM is suggested to play a role in tumor metastasis.^27^ Neoplasia including renal cancer,^28^ breast cancer,^29^ lung cancer,^30^ colon cancer,^31^ as well as melanoma^32^ is characterized by the presence of extracellular collagen fibrils and bundles that can be assessed by SHG imaging. Brain metastases are, therefore, frequently characterized by strong alterations in the ECM compared to nontumor brain.^15^ This explains why the analysis of SHG images was more suited for discrimination of secondary brain tumors than for discrimination of primary brain tumors versus non-neoplastic brain. As a drawback for the analysis of single FoVs, SHG-active structures are local features. They are present in some locations of the tumors while being absent in other regions, which might be the underlying cause why the classification of brain tumor versus nontumor brain based on analysis of SHG images was not successful. However, the surgeon or more sophisticated type of image analysis might integrate this information, as the presence of any abnormal SHG signal is an unquestionable indication of brain pathologies.^15^

Texture analysis of CARS images permitted the classifications of the tumor and nontumor brain samples with correct rates above 75% for most tumor types but low-grade astrocytoma and recurrent GBM. CARS intensity has been proven to be a reliable measure for the delineation of all types of brain tumors that have been tested so far. This is further supported by studies using Raman spectroscopy. It was shown that experimental brain tumors, human primary brain tumors, and brain metastases of peripheral cancer display decreased intensities of the C–H bond related Raman band at 2850 cm^-1^.^11,33,34^ Furthermore, CARS visualizes a variety of explicit tumor features like the presence of lipid droplets, the increased cell density, the presence of a pronounced nuclear membrane, and enlarged nuclei of tumor cells.^12,15,35^ Moreover, stimulated Raman spectroscopy (SRS) visualizing C–H bond vibrations was likewise shown to hold great potential for label-free histopathology.^36–38^ In the present study, the analysis of the CARS signal resulted in high specificity, meaning it was particularly reliable for the recognition of nontumor brain tissue. This might be due to the fact that, even if the presence of axons being more or less pronounced, CARS images of the nontumor brain had a regular, homogenous appearance within a FoV and intra- and interpatient variability was low.

The classification of texture parameters of TPEF images provided excellent accuracies for all types of brain tumors. The presence of fluorescent cells has been described for nontumor brain areas. The fluorescent compound lipofuscin accumulates in postmitotic cells, especially in large neurons.^39^ Moreover, certain tissue layers are characterized by punctuate fluorescence.^40^ Conclusively, invasive cancer and replacement of regular fluorescent brain cells by tumor cells alter the pattern of normal brain’s fluorescence. Moreover, vascular leakage might lead to diffuse fluorescence. Spectral analysis and fluorescence lifetime have already been shown to be important for discrimination of glioma, metastases, and meningioma versus brain tissue.^41,42^ Moreover, the fluorescence signal carries information on cellularity, as it was already employed to perform segmentation of cell nuclei in brain tumor samples.^16^

Here, we developed a strategy for automated tumor recognition based on label-free multiphoton imaging using a large cohort of patients and showed its potential for clinical translation by investigation of fresh biopsies. Interestingly, images with high resolution were not required for our approach. This is important, as lower resolution images can be obtained with shorter acquisition time, which is critical for any intraoperative in situ application of microscopic techniques and their clinical translation. Moreover, in situ applications require multiphoton imaging using not microscopes but endoscopic systems whose lateral resolution is limited by construction and optics. Technical solutions for simultaneous CARS and TPEF imaging in situ, before removal of suspicious tissue, are currently being developed. Multiphoton endoscopes employing long 19 cm GRIN lenses with a diameter of only 2.2 mm^43^ as well as rigid endoscopes with a diameter of 12 mm and a length of 27 cm^44^ have been successfully tested for imaging of nervous tissue in a reflection configuration. Those endoscopic systems for CARS and multiphoton (including TPEF) applications are able to provide a lateral resolution of about 1 µm^43,44^ that was sufficient for tumor recognition in our study. Their dimensions are compatible with neurosurgical applications. Therefore, the approach presented in this paper on fresh biopsies is transferrable to clinical applications, as similar FoV and lateral resolution can be provided by existing endoscopic systems. Moreover, the future development of endoscopic SRS systems might further improve intraoperative tumor diagnostics exploiting the higher biochemical specificity of SRS.^45^

Before any diagnostic routine clinical methodology can be developed, larger multicenter studies are required. The tumor variability has to be carefully analyzed to determine how many images for each location are needed to obtain a stable, reliable diagnostic result. As a first indication, our results suggest that analysis of a few images might be sufficient, because all FoVs were assigned to the correct class for most samples. Future work has to define to what degree of infiltration tumor-induced tissue changes can be detected by analysis of texture parameters and if diagnostic information can be extracted. Moreover, mathematical approaches might be refined on an extended set of fresh samples to further validate the potential of label-free multiphoton microscopy for intraoperative tumor recognition and analysis of the tumor border of different tumor types. For relevant types of brain tumors, specific classification algorithms should be developed and problem-dependent thresholds defined in order to tune the sensitivity for the detection of tumor cell infiltrations. However, texture analysis proved to be an appropriate approach for the analysis of label-free multiphoton images giving reliable results for recognition of nontumor tissue versus samples of brain tumors. We found that differentiation of nontumor brain versus many different types of neoplastic brain tissue ranging from low-grade glioma to metastases of peripheral cancers can be likewise achieved. Therefore, it constitutes an excellent approach for neurosurgical applications. Using an endoscopic instrument, in situ CARS/TPEF images of suspicious tissue can be acquired and analyzed within seconds without prior diagnostic information. Intraoperative tumor delineation can be achieved even in cases that are not suited for fluorescence guidance using 5-ALA. We expect that the additional information about tissue type will enable the neurosurgeon to better appreciate tumor margins and to adjust the extent of resection.

## Supplementary Material

vdaa035_suppl_Supplementary_Material

## Notes

**Authorship Statement** M.K., G.St., R.G., and O.U. conceived the research; G.M., O.U., and R.G. performed the experiments; O.U., R.G., and G.M. analyzed the data; M.M. provided histopathological expertise; G.Sch. and E.K. supplied research facilities; O.U. wrote the draft manuscript; R.G. and G.St. revised the draft manuscript. All authors reviewed and approved the final manuscript.
