# Supplementary material for: Label-free multiphoton imaging allows brain tumor recognition based on texture analysis—a study of 382 tumor patients
Source: Neurooncol Adv. 2020 Mar 12;2(1):vdaa035. doi: 10.1093/noajnl/vdaa035 (PMC7212881; doi:10.1093/noajnl/vdaa035)
Supplement: vdaa035_suppl_Supplementary_Material [file vdaa035_suppl_supplementary_material.pdf]

Supplementary Materials:

Label-free multiphoton imaging allows brain tumor recognition based on texture analysis – a study of 382 tumor patients

Ortrud Uckermann, Roberta Galli, George Mark, Matthias Meinhardt, Edmund Koch, Gabriele Schackert, Gerald Steiner, Matthias Kirsch

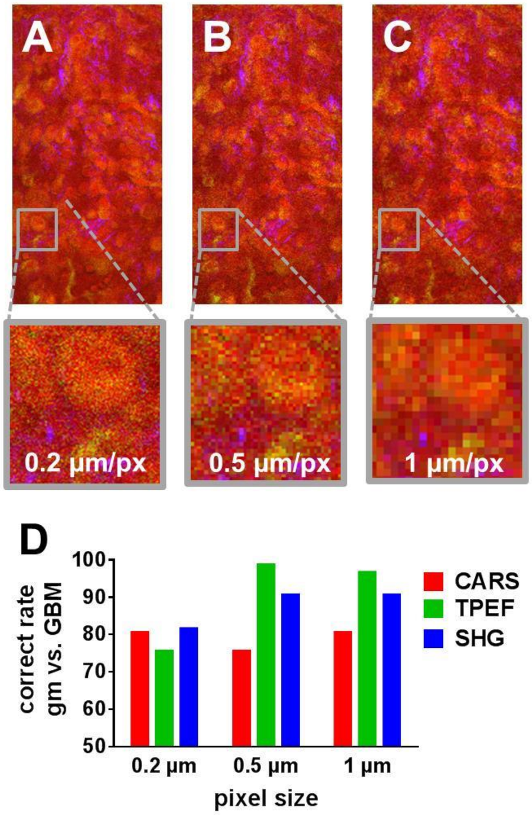

Supplementary Figure S1. Influence of image resolution on classification result. A-C: CARS/TPEF/SHG images of GBM (rehydrated cryosection, field of view 104 x 208 µm) acquired with pixel size of 0.2 µm, 0.5 µm and 1 µm. Magnification of the area in the box is shown in the lower part **D:** Correct rate of the classification of images of non-tumor gray matter (gm, n=40) versus GBM (n=38) for the different pixel sizes for each channel. Classification using linear discriminant analysis (MATLAB function 'classify') with leave-one-out approach was used. ~80% of images were correctly classified based on the texture parameters extracted from the CARS channel, irrespective of image resolution (81%, 76%, 81%). The correct rate for the TPEF and SHG channels was similar or even higher for images with larger pixel size (TPEF: 76%, 99%, 97%; SHG: 82%, 91%, 91%).

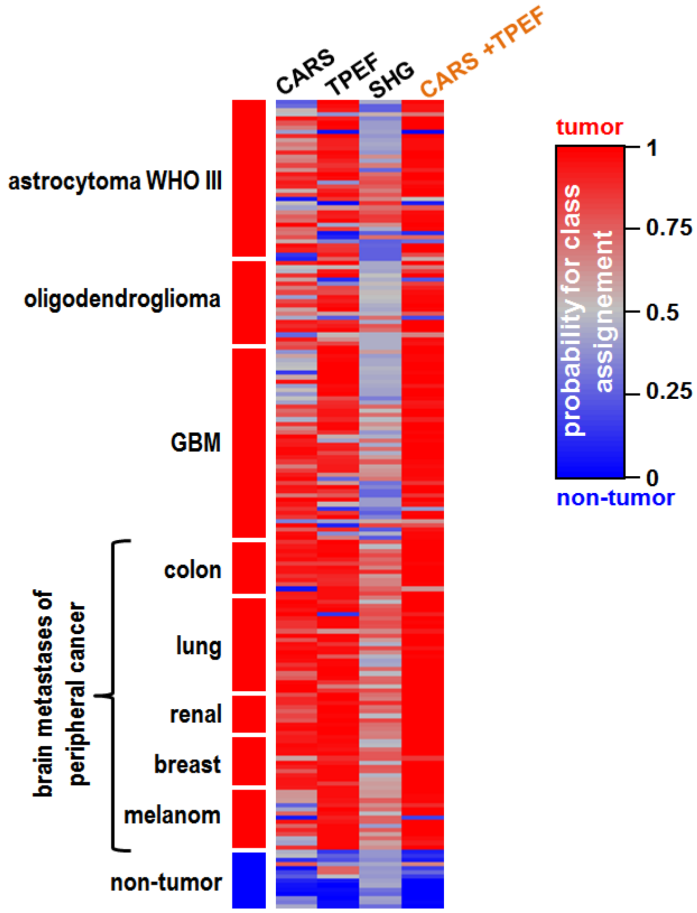

Supplementary Figure S2. Reclassification of the training set for primary and secondary brain tumors versus non-tumor brain tissue. The probability of class assignment is plotted each patient of the training set using a color code ranging from red (tumor) to blue (non-tumor). Linear discriminant analysis of texture parameters of brain tumors (n=177) versus non-tumor brain tissue (n=14) was performed. Results based on analysis of CARS, TPEF and SHG images and the combination of CARS+TPEF.

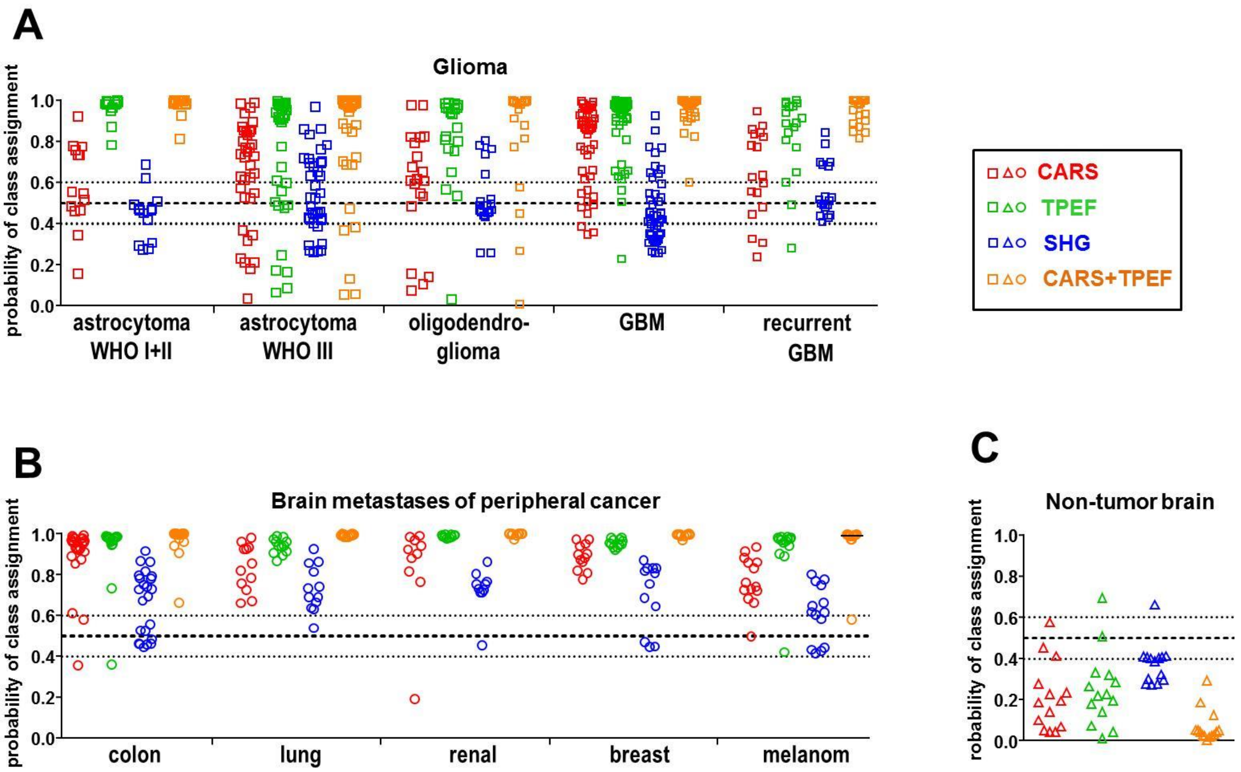

Supplementary Figure S3. Classification result for different types of brain tumors versus non-tumor brain tissue. A: Gioma **B:** Brain metasates of peripheral cancers **C:** non-neoplastic brain. The probability of class assignment is plotted each for patient of the test set for CARS (red symbols), TPEF (green symbols) and SHG images (blue symbols) and combined analysis of CARS+TPEF images, respectively. Linear discriminant analysis of texture parameters was performed using an independent training set build from images of the respective type of glioma and non-tumor brain tissue (Astocytoma WHO I/II n=14, anaplastic astrocytoma WHO III n=36, anaplastic oligodendroglioma WHO III n=20, GBM WHO IV n=45, recurrent GBM WHO IV n=18, brain metastasis of lung cancer n=23, of colon cancer n=12, of renal cancer n=10, of breast cancer n=12, of malignant melanoma n=15, non-tumor brain tissue n=14).

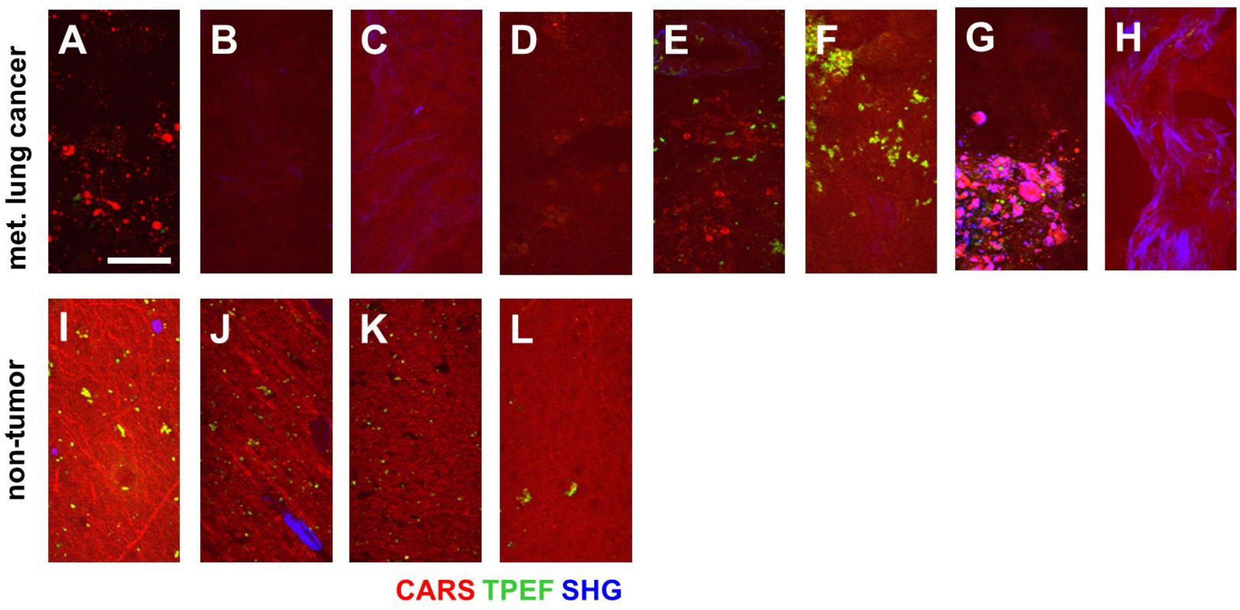

Supplementary Figure S4. Raw images of the examples of human metastases of lung cancer and non-tumor tissue. (**A-H**): Label-free multiphoton images of metastases of lung cancer. **(I-L)**: Label-free multiphoton images of non-tumor brain tissue, scale bar: 50 µm.

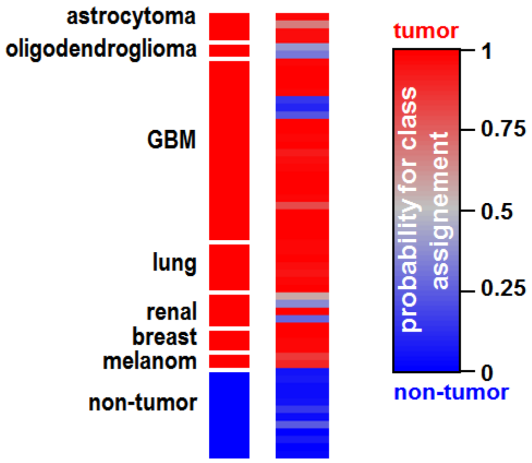

Supplementary Figure S5. Reclassification of the training set for fresh brain tumor biopsies versus non-tumor brain tissue. The Probability of class assignment is plotted for each sample of the training set using a color code ranging from red (tumor) to blue (non-tumor). Linear discriminant analysis of texture parameters of brain tumor biopsies (n=47 samples of 12 patients) versus non-tumor brain tissue (n=12 samples of 9 patients/body donors) was performed. Results based on combined analysis of CARS+TPEF images.

Supplementary Table S1. Composition of test and training set. The number of patients is given for each tissue type. The number of samples is given in parenthesis for fresh tissue

Tissue type		cryosections		fresh tissue	
	total	test	training	test	training
low grade astrocytoma WHO I/II	14	14			
anaplastic astrocytoma WHO III	73	36	37	1(5)	1(4)
anaplastic oligodendroglioma WHO III	41	20	21		1(2)
GBM	91	45	46	6(20)	6(24)
recurrent GBM	18	18			
Brain metastases of					
lung cancer	46	23	23	1(2)	1(7)
colon cancer	25	12	13		
renal cancer	20	10	10		1(5)
breast cancer	24	12	12	1(4)	1(3)
prostate cancer	1			1(1)	
malignant melanoma	29	14	15	1(3)	1(2)
Non-neoplastic	28	14	14	10	9(12)
